# Transferring Exome Sequencing Data from Clinical Laboratories to Healthcare Providers: Lessons Learned at a Pediatric Hospital

**DOI:** 10.3389/fgene.2018.00054

**Published:** 2018-02-21

**Authors:** Rajeswari Swaminathan, Yungui Huang, Katherine Miller, Matthew Pastore, Sayaka Hashimoto, Theodora Jacobson, Danielle Mouhlas, Simon Lin

**Affiliations:** ^1^Research Information Solutions and Innovation, The Research Institute at Nationwide Children's Hospital, Columbus, OH, United States; ^2^Division of Molecular and Human Genetics, Nationwide Children's Hospital, Columbus, OH, United States; ^3^Institute for Genomic Medicine, Nationwide Children's Hospital, Columbus, OH, United States

**Keywords:** genomic data sharing, genomic data transfer, whole exome sequencing, clinical genomics, interoperability, laboratory workflows

## Abstract

The adoption rate of genome sequencing for clinical diagnostics has been steadily increasing leading to the possibility of improvement in diagnostic yields. Although laboratories generate a summary clinical report, sharing raw genomic data with healthcare providers is equally important, both for secondary research studies as well as for a deeper analysis of the data itself, as seen by the efforts from organizations such as American College of Medical Genetics and Genomics and Global Alliance for Genomics and Health. Here, we aim to describe the existing protocol of genomic data sharing between a certified clinical laboratory and a healthcare provider and highlight some of the lessons learned. This study tracked and subsequently evaluated the data transfer workflow for 19 patients, all of whom consented to be part of this research study and visited the genetics clinic at a tertiary pediatric hospital between April 2016 to December 2016. Two of the most noticeable elements observed through this study are the manual validation steps and the discrepancies in patient identifiers used by a clinical lab vs. healthcare provider. Both of these add complexity to the transfer process as well as make it more susceptible to errors. The results from this study highlight some of the critical changes that need to be made in order to improve genomic data sharing workflows between healthcare providers and clinical sequencing laboratories.

## Introduction

The rate of genome sequencing is rising sharply, leading to the generation of humungous volumes of data. Despite the surge in data generation, utilizing the wealth of knowledge embedded in that data for the improvement of clinical outcomes is still lagging behind (Ginsburg, 2014). Additional research is still required in order to better associate genes/variants with diseases. Currently, clinical laboratories return a summary report back to the ordering physician. However, depending on the complexity of the disease as well as the availability of information within knowledge bases, not every report ends up with a diagnosis. In many cases, when a sequencing rest is unable to detect the underlying genetic cause, clinicians may choose to obtain the raw sequencing data (available as FASTQ, VCF, or BAM files) and perform a more detailed research study/analysis on it, in hopes of untangling some of the complex details associated with the case. However, the underlying decision to share data ultimately rests in the hands of the patient/participant. Sharing sequencing data directly with the patient itself can also be beneficial, especially when a researcher does not have adequate resources to return any clinically actionable information back to the patient (Middleton et al., 2015). Sharing data directly with individuals makes them feel empowered and better control the further flow of their confidential information (Shabani et al., 2017). There are currently several initiatives, such as GenomeConnect, My Research Legacy by the American Heart Association, etc. that are involved in sharing biomedical information for research and health purposes (Miller and Lin, 2017). Although there are several challenges associated with patient controlled sharing of genomic data, it is not within the scope of the current study.

At present, clinical laboratories either load the data onto hard drives/Universal Serial Bus (USB) drives and ship them to the providers or directly transfer data over a secure network. There is currently no standard protocol for transferring sequencing data from laboratories to healthcare providers. Through this study, we aim to describe the current state of the genomic data transfer process, specifically, data obtained from WES studies between sequencing laboratories and healthcare providers and highlight some of the key lessons learned.

## Materials and methods

**Figure 1:**
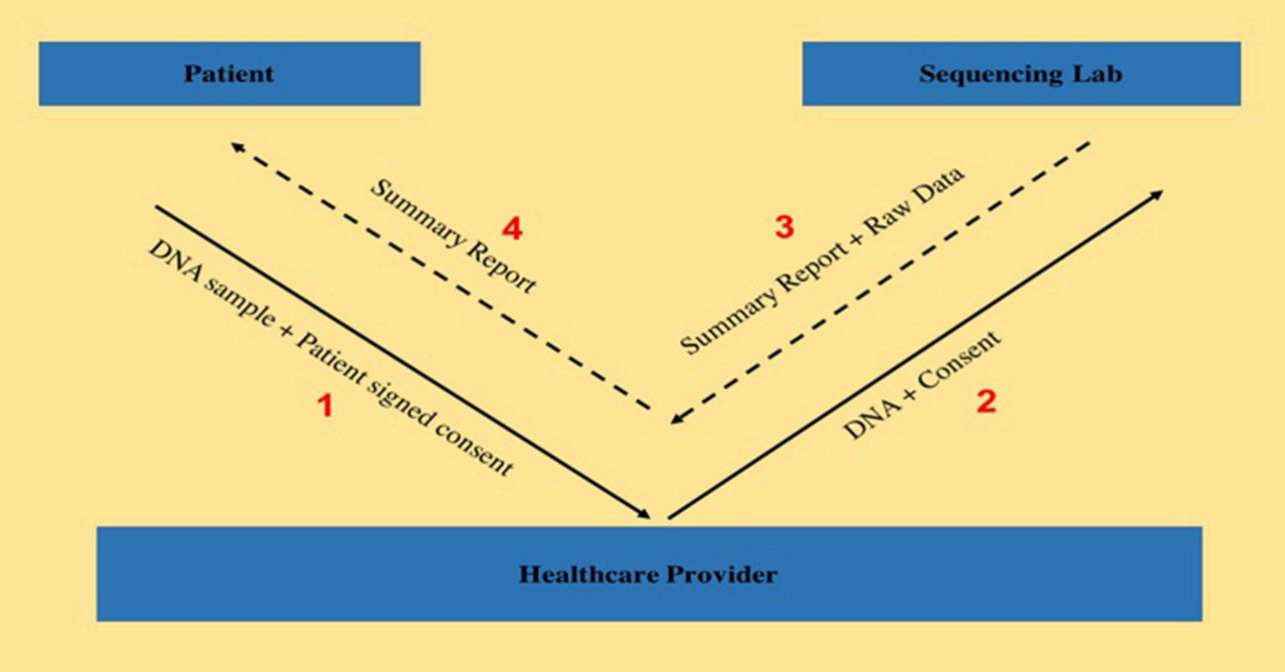
This figure shows the different steps and entities involved in the process that starts from a patient consenting for WES and release of sequencing data, to the sequencing being performed in the sequencing lab and finally releasing the test result as well as transferring the raw sequencing data (FASTQ file) to the healthcare provider.

During the observation period of this study from April 2016 to December 2016, samples from 122 patients admitted to a tertiary pediatric hospital and ordered for WES testing were sent to a genetic laboratory accredited by CAP and certified by CLIA. Since genomic data is considered private and confidential, explicit consent had to be obtained from the patients in order to be able to use their data for research purposes. Nineteen of the 122 patients provided consent to have their WES data transferred from the laboratory to the researchers associated with the provider institution. There are many reasons for not being able to obtain patient consent, starting with participants having a complete lack of interest in research all the way to having to face discriminatory treatment in the event of being diagnosed with a high risk disease mutation. The workflow, as shown in Figure 1 below, describes the steps involved from consenting the patient to receiving data back from the laboratory. For all 19 patients, the consent for WES as well as for raw data release were obtained on the same day by the same provider. Turnaround time for WES report release is approximately 12 weeks. Once the report is released, the raw data is independently released by the laboratory.

For securely transferring large volumes of health data, the laboratory in this study uses a “Managed File Transfer System” (MFTS), a service providing fine-grained access and control features over using simple Secure File Transfer Protocol (SFTP) clients^1^ The MFTS service uses both SFTP and HyperText Transfer Protocol Secure (HTTPS) protocols underneath for performing data transfers, and users can download the data through either client. The FASTQ files are deposited on a laboratory server, where they stay up to 90 days, from the date of upload. The laboratory sends a notification to the provider email address listed on the Data Release consent form. Validation is performed by comparing the identifier on the notification with the identifier listed on the Data Release form to ensure integrity of the data being downloaded. Healthcare providers are given a secure login-based access to a restricted section on the server, containing only the data from their consented patients.

## Results

As seen in Table 1, the time taken by the laboratory to process each of the data release requests varied considerably. The “–” in some places is due to the missing information on some of the WES report release dates. The average turnaround time from the time of test report release to having the raw data ready for download was around 9.7 weeks, with a maximum of 26 weeks, minimum of 1 week and standard deviation of 8.5 weeks. The huge difference in processing times in the early cases compared to those toward the end can be attributed to the improvement in process workflow along the course of this study. When the study began, there was no standardized process in place for sending files over from the laboratory to the healthcare provider. Further, there were no protocols in place for creating specific users for the healthcare provider to access and download the data. However, as the process was repeatedly applied on subsequent cases, there was an iterative improvement to the workflow as can be seen by the significant decrease in processing times.

**Table 1:** Time taken from sending consent form to having data ready for download for each of the 19 patients in the study.

**Patient ID**	**Date data release consent form sent to sequencing lab**	**Time from consent sent to raw data release (in weeks)**	**Time from WES result release to FASTQ data release (in weeks)**
1	4/18/16	34	–
2	4/21/16	34	26 weeks
3	4/15/16	35	21 weeks
4	5/16/16	31	21 weeks
5	6/6/16	16	2.5 weeks
6	6/30/16	24	14 weeks
7	6/17/16	15	6 weeks
8	6/29/16	24	17 weeks
9	6/28/16	16	1 week
10	6/24/16	25	12 weeks
11	7/6/16	23	14.5 weeks
12	5/19/16	30	17 weeks
13	7/15/16	12	1 week
14	8/22/16	11	1.5 week
15	8/2/16	19	6 weeks
16	7/13/16	17	1 week
17	7/11/16	22	1.5 weeks
18	8/10/16	18	2 weeks
19	9/23/16	10	–

Paper-based patient consents obtained by the genetic counselors are physically sent to the genetic laboratory along with the blood or DNA sample, printed medical records, and other appropriate information. We observed challenges in consistently providing all of the required information to the laboratory. One of the challenges to this manual process is the possibility of dealing with missing information. There were two cases in the current study, where patient consent forms were missing, but the data was available for download. On the other hand, there was a single case of a patient who provided consent, but there was no data available for download. Each time the data is available for download, a manual notification needs to be sent by the laboratory personnel to the provider, alerting them of the availability of data, which can lead to unnecessary wait times. Thirdly, there are discrepancies between the provider and the laboratory in uniquely identifying a sample. In this study, the consent forms by the provider used patient name and DOB, but the sequencing lab assigned a DNA sample number to uniquely identify each patient in the data download notification. One of the Data Release forms did mention the DNA Sample Number, but the others used the combined Patient Name + DOB. The email notifications sent by the sequencing lab notifying the healthcare provider that the FASTQ files are ready for download also uses the DNA sample number as the identifier. It is necessary to verify the DNA sample number in the data download notification matches with the identifier on the consent forms to make sure only data with appropriate consents are being transferred, thereby introducing an additional mapping step. Although the workflow became more robust and the processing times reduced significantly toward the end of the study, the process is not completely free of manual interferences.

## Discussion

The results from this study highlight an urgent need to implement automated systems to improve information exchange between healthcare providers and clinical genetic laboratories. As stated by the American College of Medical Genetics and Genomics (ACMG), genomic data sharing is extremely important for the development of new diagnostic techniques and therapeutics that will ultimately lead to an improvement of patient care and understanding of disease (Acmg Board Of D, 2017). The importance of genomic data and its impact on health outcomes is also entering the minds of patients now. Since the ultimate owner of the data are the patients themselves, it is important that they realize this need in order to provide the required consent. Repeated sessions of genetic counseling and the widespread information available on the internet have helped educate patients to a considerable extent (Morgan et al., 2014). Having manual control of a possibly frequently used process in the future can lead to unwanted errors. Using the electronic health record (EHR) system to store all this data comes with the advantage that triggers could be set in place to validate all of the incoming and outgoing data as well as send automated notifications. On a shared note, since patients often see multiple healthcare providers during their lifetime and have their data shared across multiple provider institutions, an interoperable Application Programming Interface (API) connecting the different systems would also be required in the future. This will eliminate the hassle of writing individual programs for each of the data access requests. In order to access genomic data across multiple systems, existing consortiums such as the Global Alliance for Genomics and Health (GA4GH), provide an interoperable genomics framework that can be accessed through an API (Global Alliance for Genomics and Health, 2016; Swaminathan et al., 2016). Additionally, the Office of the National Coordinator for Health Information Technology (ONC) encourages those involved in health IT to contribute to the development of a defined, shared, roadmap leveraging health IT interoperability to ultimately protect and advance healthcare for all (Technology, 2018).

Similar to how all research sequencing data is stored in the centralized repository, dbGap (Tryka et al., 2014), sequencing laboratories can also deposit all of the clinical sequencing data into a similar centralized location and later provide appropriate access to researchers. The genomic world is also looking into the possibility of using a blockchain framework for the seamless sharing of sensitive genomic information. Instead of sharing data with the healthcare providers, who would eventually pass it on to the research community, the sequencing laboratories can also consider sharing the data directly with the patient themselves, who own that data. This way even if the data needs to be shared with multiple researchers, it can be taken care of by the patient themselves.

The current methods of secure data transfer, mainly by shipping hard drives. can be costly to providers (~150–200 USD). One prospective option is to store data in a centralized cloud and provide access to interested parties in a secure manner. Although the concept of the Health Insurance Portability and Accountability Act (HIPAA)-compliant clouds is slowly coming into existence, maintaining security and privacy of genomic data in the cloud still remains an outstanding question for many organizations.

In conclusion, there is massive potential to leverage genomic data to advance human health overall. The medical community needs to be able to share genomic data to achieve better and improved patient outcomes. Our study highlights some of the hurdles that can be encountered and some potential ways to address them in order to achieve the path to successful implementation of secure and efficient genomic data transfer and sharing.

## Data

All 19 patients whose data has been used as part of this study consented for their data to be used for research studies. Since this is just a Quality Improvement (QI) project, there was no requirement to pass through the ethics committee. There was no analysis or manipulation done to data from any of the patients.

## Author contributions

RS, YH, and SL conceived and designed the study. MP, SH, TJ, and DM obtained consent from patients and worked on obtaining the required data for the study. RS, YH, KM, and SL drafted the manuscript. All authors read, edited and approved the final manuscript as written.

### Conflict of interest statement

The authors declare that the research was conducted in the absence of any commercial or financial relationships that could be construed as a potential conflict of interest.

## Footnotes

^1^*Explanation of the FTP and SFTP protocols*. Available online at: http://www.wise-ftp.com/know-how/ftp_and_sftp.htm.

## Abbreviations

WES: Whole Exome Sequencing
CAP: College of American Pathologists
CLIA: Clinical Laboratory Improvement Amendments
ACMG: American College of Medical Genetics and Genomics
EHR: Electronic Health Record
MFTS: Managed File Transfer System
SFTP: Secure File Transfer Protocol
HTTPS: HyperText Transfer Protocol Secure
DOB: Date of Birth
MRN: Medical Record Number
GA4GH: Global Alliance for Genomics and Health
API: Application Programming Interface
HIPAA: Health Insurance Portability and Accountability Act
ONC: Office of the National Coordinator for Health Information Technology.

## References

[B1] Acmg Board Of D (2017). Laboratory and clinical genomic data sharing is crucial to improving genetic health care: a position statement of the American College of Medical Genetics and Genomics. Genet. Med. 19, 721–722. 10.1038/gim.2016.196 28055021

[B2] Ginsburg G. (2014). Medical genomics: gather and use genetic data in health care. Nature 508, 451–453. 10.1038/508451a 24765668

[B3] Global Alliance for Genomics and Health (2016). GENOMICS. A federated ecosystem for sharing genomic, clinical data. Science 352, 1278–1280. 10.1126/science.aaf6162 27284183

[B4] Middleton A. Wright C. F. Morley K. I. Bragin E. Firth H. V. Hurles M. E. (2015). Potential research participants support the return of raw sequence data. J. Med. Genet. 52, 571–574. 10.1136/jmedgenet-2015-103119 25995218 PMC4518751

[B5] Miller K. E. Lin S. M. (2017). Addressing a patient-controlled approach for genomic data sharing. Genet. Med. 19, 1280–1281. 10.1038/gim.2017.36 28425983

[B6] Morgan T. Schmidt J. Haakonsen C. Lewis J. Della Rocca M. Morrison S.. (2014). Using the internet to seek information about genetic and rare diseases: a case study comparing data from 2006 and 2011. JMIR Res. Protoc. 3:e10. 10.2196/resprot.2916 24565858 PMC3961701

[B7] Shabani M. Vears D. Borry P. (2017). Raw genomic data: storage, access, and sharing. Trends Genet. 34, 8–10. 10.1016/j.tig.2017.10.004 29132689

[B8] Swaminathan R. Huang Y. Moosavinasab S. L. Buckley R. Bartlett C. W. Lin S. M. (2016). A Review on Genomics APIs. Comput. Struct. Biotechnol. J. 14, 8–15. 10.1016/j.csbj.2015.10.004 26702340 PMC4669666

[B9] Technology, T. O. o. t. N. C. f. H. I. (2018). Connecting Health and Care for the Nation. Available online at: https://www.healthit.gov/sites/default/files/hie-interoperability/nationwide-interoperability-roadmap-final-version-1.0.pdf

[B10] Tryka K. A. Hao L. Sturcke A. Jin Y. Wang Z. Y. Ziyabari L.. (2014). NCBI's database of genotypes and phenotypes: dbGaP. Nucleic Acids Res. 42, D975–D979. 10.1093/nar/gkt1211 24297256 PMC3965052

